# Characterization of Three Venom Peptides from the Spitting Spider *Scytodes thoracica*

**DOI:** 10.1371/journal.pone.0156291

**Published:** 2016-05-26

**Authors:** Nathanial K. Ariki, Lisa E. Muñoz, Elizabeth L. Armitage, Francesca R. Goodstein, Kathryn G. George, Vanessa L. Smith, Irina Vetter, Volker Herzig, Glenn F. King, Nikolaus M. Loening

**Affiliations:** 1 Chemistry Department, Lewis & Clark College, 0615 SW Palatine Hill Road, Portland, OR, 97219, United States of America; 2 Institute for Molecular Bioscience, The University of Queensland, St. Lucia, QLD 4072, Australia; Weizmann Institute of Science, ISRAEL

## Abstract

We present the solution-state NMR structures and preliminary functional characterizations of three venom peptides identified from the spitting spider *Scytodes thoracica*. Despite little sequence identity to other venom peptides, structural characterization reveals that these peptides contain an inhibitor cystine knot motif common to many venom peptides. These are the first structures for any peptide or protein from spiders of the Scytodidae family. Many venom peptides target neuronal ion channels or receptors. However, we have not been able to determine the target of these *Scytodes* peptides so we can only state with certainty the channels and receptors that they do not target.

## Introduction

Spider venoms are cocktails of peptides, proteins, and small organic molecules [1,2], with peptides being the most abundant compound. Each species of spider can produce hundreds or thousands of different venom peptides, which suggests that the number of unique venom peptides could be upwards of 12 million [3]. However, to date, researchers have characterized only a small fraction of these peptides; some 1403 at last count [4]. A handful of those characterized are potent modulators of neuronal ion channels and receptors [5–7]. While spiders primarily prey on insects, these venom peptides can target a wide range of invertebrate and vertebrate ion channels, including those found in humans and other mammals [1].

Disulfide-rich peptides are the dominant components in most spider venoms and they are often the key contributors to the activity and potency of the venom [8]. While these disulfide-rich peptides can adopt a number of different structural motifs, the motif known as the inhibitor cystine knot (ICK) is the most widely observed. ICK peptides contain an antiparallel β-sheet stabilized by three or more disulfide bonds, creating a knot in the core of the peptide [9]. The consensus sequence for the ICK motif [10] is:

$${\text{C}}_{\text{I}}{\text{X}}_{3-7}{\text{C}}_{\text{II}}{\text{X}}_{3-8}{\text{C}}_{\text{III}}{\text{X}}_{0-7}{\text{C}}_{\text{IV}}{\text{X}}_{1-6}{\text{C}}_{\text{V}}{\text{X}}_{3-13}{\text{C}}_{\text{VI}}$$

where “C” represents one of the six cysteines in the motif and “X” represents a stretch of other amino acid residues. The number of amino acid residues between cysteines varies, but is typically within the ranges given in the subscripts. The cystine knot is comprised of a ring formed between two disulfide bonds (C_I_–C_IV_, C_II_–C_V_) and the peptide backbone, with a third disulfide bond (C_III_–C_VI_) penetrating the ring. This pseudo-knot confers these venom peptides with remarkable chemical and thermal stability; they are resistant to extremes of temperature and pH and have shown resistance to proteolytic degradation [11], even within gastric environments [1].

The repeated use of the ICK motif in spider venom presumably reflects the effectiveness of this structural class for generating large assortments of functionally diverse and stable peptides that can target a wide array of molecular targets. Gene duplication and diversification of the peptide sequence surrounding the knotted core has allowed this ICK structure to act as an adaptable framework for a wide range of peptide sequences [12] that can target neuronal ion channels with relative selectivity and potent paralytic or lethal function [13]. Due to their robustness and target specificity, ICK peptides are promising candidates for use in preventing agricultural crop loss due to insect pests [8,14], and for use as therapeutic modulators of ion channels in humans [1].

In this paper, we present the structures of three venom peptides from the spider *Scytodes thoracica*. *Scytodes* are known as “spitting spiders” due to their unusual hunting method; they first restrain their prey with gluey spit before approaching and injecting venom to further immobilize their prey [15]. The three structures that we determined are, to date, the only peptide or protein structures from Scytodidae, a family that includes five genera and 232 species [16]. In addition to these structures, we present work towards the functional characterization of these peptides. Unfortunately, despite performing injections and topical applications of recombinantly-produced peptides into insects, fluorescent assays with ion channels, and radioligand screening against central nervous system receptors, we have not yet been able to determine the targets of these peptides.

## Methods

### mRNA Identification and Sequence Analysis

The sequences that we studied were first identified by Binford and colleagues using cDNA libraries generated from venom gland mRNA [15]. The resulting data set contained more than 50 sequences that had a high likelihood of being venom toxins based on sequence homology and the pattern of cysteine residues.

**Fig 1:**
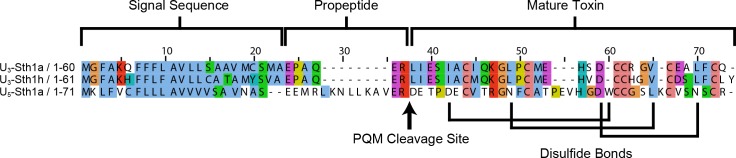
Sequence alignment for the translated sequences of U_3_-Sth1a, U_3_-Sth1h, and U_5_-Sth1a. Alignment of *Scytodes* venom peptides illustrating the signal sequence, propeptide, and mature toxin regions. The signal sequence predicted by SignalP [21] is two residues shorter for U_5_-Sth1a than the other two peptides. The arrow after the processing quadruplet motif (PQM) [27] indicates the predicted cleavage sites for the mature toxins. The experimentally-determined disulfide-bond connectivity is shown below the alignment. Sequences were aligned using ClustalX 2.1 [51] and visualized using JalView 2.8.1 [52]. The coloring makes use of the default ClustalX color scheme, which is a function of sequence identity and amino acid type.

From this set of putative venom toxin sequences, we selected three for further study based on the amount of peptide produced using our bacterial expression system. These three peptides, which had been given the names U_3_-scytotoxin-Sth1a, U_3_-scytotoxin-Sth1h, and U_5_-scytotoxin-Sth1a following the proposed unified nomenclature for spider toxins [17], will be for purposes of brevity referred to as U_3_-Sth1a, U_3_-Sth1h, and U_5_-Sth1a, respectively, in the following text. The full sequences for U_3_-Sth1a, U_3_-Sth1h, and U_5_-Sth1a are shown in Fig 1 and the UniProt accession numbers are A0A0A0V662, A0A0A0V712, and A0A0A0V633, respectively (see Table 1).

**Table 1:** Accession Numbers for U_3_-Sth1a, U_3_-Sth1h, and U_5_-Sth1a.

	U_3_-Sth1a	U_3_-Sth1h	U_5_-Sth1a
Full Name	U_3_-scytotoxin-Sth1a	U_3_-scytotoxin-Sth1h	U_5_-scytotoxin-Sth1a
UniProt Accession Number	A0A0A0V662	A0A0A0V712	A0A0A0V633
BioMagResBank Number	26002	26003	26004
Protein Data Bank Code	5FZV	5FZW	5FZX
ArachnoServer Code	AS001422	AS001429	AS001474

Venom peptides are typically expressed as prepropeptides containing a conserved N-terminal hydrophobic α-helical signaling sequence, a mature toxin sequence at the C-terminal end, and a propeptide region connecting the two. The N-terminal signaling sequence directs the translation of the prepropeptide into the lumen of the endoplasmic reticulum [1,18]. Subsequent proteolytic cleavage steps remove the N-terminal signaling sequence and propeptide, leaving only the mature toxin sequence. This mature toxin sequence then usually folds without any further post-translational modifications into an active ICK peptide [19,20].

For the three *Scytodes* peptide sequences, we predicted the location of the signal sequences indicated in Fig 1 using SignalP 4.0 [21]. As is typical, the signal sequences are rich in hydrophobic amino acids (shown colored blue). The location of the cleavage site that results in the mature toxin is shown by an arrow in Fig 1. This cleavage site was predicted using the consensus sequence for the processing quadruplet motif (PQM) [22]. In the consensus sequence for the PQM:

$${\text{X}}_{-4}{\text{X}}_{-3}{\text{X}}_{-2}{\text{R}}_{-1}\text{Y}$$

where Y is the first amino acid of the mature toxin sequence, the cleavage site for the mature toxin is immediately after the arginine (R), and at least one of the three amino acids prior to the arginine (labeled X) is a glutamate (E). For U_5_-Sth1a, there is another match to the PQM consensus sequence immediately after the signal sequence. However, sequence alignment with other known full-length spider venom toxin sequences indicates that cleavage at this site would result in an uncharacteristically short linker sequence. In addition, proteomics work by Binford and colleagues using mass spectrometry [15] confirmed the presence of U_5_-Sth1a with the cleavage pattern indicated in Fig 1 in crude *Scytodes thoracica* venom.

### Vector Design for Recombinant Expression

Codon-optimized genes encoding each of the mature toxins were synthesized and inserted into a variant of the pLic-MBP expression vector [23] by GeneArt (Invitrogen). The expression product from this system is a fusion protein with a signal sequence that directs transport of the fusion protein to the periplasm, a His_6_ tag, and maltose binding domain tag at the N-terminus of the toxin peptide. To allow for cleavage of the mature peptide from the rest of the fusion protein, we included a cleavage site for tobacco etch virus (TEV) protease immediately before the toxin sequence. As the mature toxin sequence for U_5_-Sth1a begins with an aspartate, it was possible to engineer the TEV protease cleavage site for the U_5_-Sth1a construct without introducing a non-native amino acid to the mature toxin sequence. For U_3_-Sth1a and U_3_-Sth1h, however, providing a TEV protease cleavage site necessitated the inclusion of a non-native glycine residue at the N-terminus of the mature toxin sequences. Consequently, the numbering of the amino acid sequences for the structures of U_3_-Sth1a and U_3_-Sth1h begins at zero rather than at one.

### U_3_-Sth1a and U_3_-Sth1h Peptide Expression and Purification

U_3_-Sth1a and U_3_-Sth1h samples were generated using a modified high-density expression protocol [24]. First, pLICC vector containing either the U_3_-Sth1a or the U_3_-Sth1h insert was used to transform *Escherichia coli* BL21 (DE3) cells. The transformed cells were incubated overnight at 37°C in Luria-Bertani (LB) medium with ampicillin (100 μg/ml) and used to generate a glycerol stock that was stored at –70°C. A scraping of the resulting glycerol stock was used to inoculate a 1 mL starter culture of LB medium containing ampicillin (100 μg/ml), and grown overnight at 37°C. This starter culture was used to inoculate a 1 L baffled flask containing 250 mL of ZYP-5052 media [25] containing carbenicillin (60 μg/ml). The ZYP-5052 media was adjusted to pH 8 prior to inoculation. The cell culture was allowed to grow at 37°C for approximately 12 h at which point the culture reached an OD_600_ of 5–7.

The cells were then spun down at 2500 rpm for 15 min and resuspended in 50% of the initial volume in a minimal media [24] that had been adjusted to pH 8 and contained carbenicillin (60 μg/ml). This medium contained ^15^N NH_4_Cl as the nitrogen source and either unlabeled glucose or ^13^C_6_-glucose as the carbon source. After 1 h of growth, the cells were induced by adding enough isopropyl β-d-1-thiogalactopyranoside (IPTG) to reach a concentration of 40 μM in the culture. The cells were incubated at 22°C for 12 h after induction and then harvested by centrifugation at 4000 rpm for 30 min.

The pellets were resuspended in a lysis buffer containing 20 mM phosphate buffer (pH 7.4), 20 mM imidazole, and 500 mM sodium chloride. After lysis by ultrasonication, the sample was centrifuged at 18,000 rpm at 4°C for 30 min. The resulting supernatant was purified using immobilized metal ion affinity chromatography (IMAC) with a HisTrap FF 5 mL column (GE Life Sciences) on an ÄKTAprime plus chromatography system (GE Life Sciences). After eluting the fusion protein from the column and buffer exchange using an Amicon centrifugal filter unit (Millipore), we cleaved the purified fusion protein overnight at room temperature using TEV protease in a redox buffer of 0.6 mM reduced glutathione (GSH)/0.4 mM oxidized glutathione (GSSG).

The cleavage reaction mixture was then fractionated using reverse-phase liquid chromatography with a Jupiter C18 column (Phenomenex) and a water/acetonitrile gradient. Fractions containing U_3_-Sth1a and U_3_-Sth1h eluted at 40% acetonitrile. Chromatography fractions were dried under vacuum and then rehydrated them in a buffer appropriate for NMR spectroscopy (95% H_2_O/5% D_2_O/20 mM sodium phosphate pH 6.5/30 mM sodium chloride). Yields of fusion protein were as high as 150 mg per liter of culture, resulting in 2–3 mg of venom peptide after the final purification step.

### U_5_-Sth1a Peptide Expression and Purification

The expression of U_5_-Sth1a followed a procedure that we previously described [26]. Briefly, the sample was prepared as follows: pLICC vector containing the U_5_-Sth1a insert was used to transform *E*. *coli* BL21 (DE3) cells, which were incubated at 22°C for 2–3 days using either a ^15^N-labeled or a ^13^C,^15^N-labeled autoinducing minimal medium [25]. The cells were then centrifuged and the resulting pellet was lysed using ultrasonication. After ultracentrifugation, the resulting supernatant was purified using IMAC. The fraction containing the fusion protein was buffer exchanged, cleaved with TEV protease at room temperature overnight in a redox buffer containing 0.6 mM GSH and 0.4 mM GSSG. This cleavage reaction mixture was then fractionated using reverse-phase liquid chromatography with a water/acetonitrile gradient. Fractions containing U_5_-Sth1a eluted at 31% acetonitrile. Chromatography fractions containing U_5_-Sth1a were dried under vacuum and then rehydrated them in a buffer appropriate for NMR spectroscopy (95% H_2_O/5% D_2_O/20 mM sodium phosphate pH 6.5/30 mM sodium chloride). Typical yields of U_5_-Sth1a fusion protein were 100–150 mg for a 0.5 L culture, resulting in 1–2 mg of venom peptide after the final purification step.

### Mass Spectrometry

**Fig 2:**
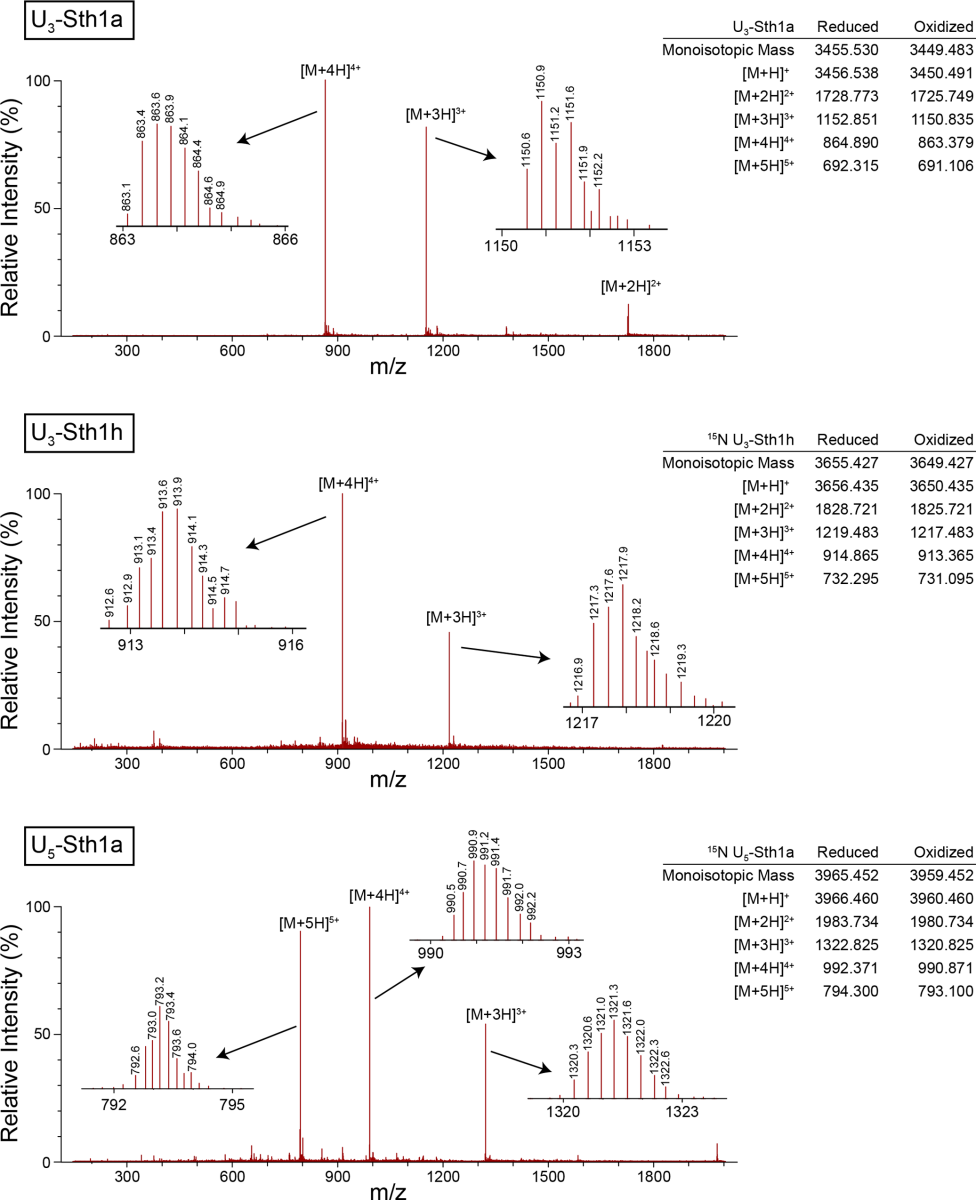
**Electrospray ionization mass spectra of U**_**3**_**-Sth1a (top), U**_**3**_**-Sth1h (middle), and U**_**5**_**-Sth1a (bottom).** Inset spectra are high-resolution zoom scans for the indicated peaks. The monoisotopic masses (in amu) and calculated mass-to-charge ratios (m/z) for the reduced and oxidized peptides are provided on the right. The sample used for U_5_-Sth1a was unlabeled whereas the samples used for U_3_-Sth1h and U_5_-Sth1a were ^15^N-labeled. The mass-to-charge ratios are consistent with fully-oxidized peptides (i.e., formation of all three disulfide bonds).

Mass spectra (Fig 2) were acquired on a Thermo Scientific Velos ion trap mass spectrometer with electrospray ionization. The sample used for U_3_-Sth1a was unlabeled whereas the samples used for U_3_-Sth1h and U_5_-Sth1a were ^15^N-labeled. The mass-to-charge ratios are consistent with fully-oxidized peptides (i.e., formation of all three disulfide bonds).

### NMR Spectroscopy

**Fig 3:**
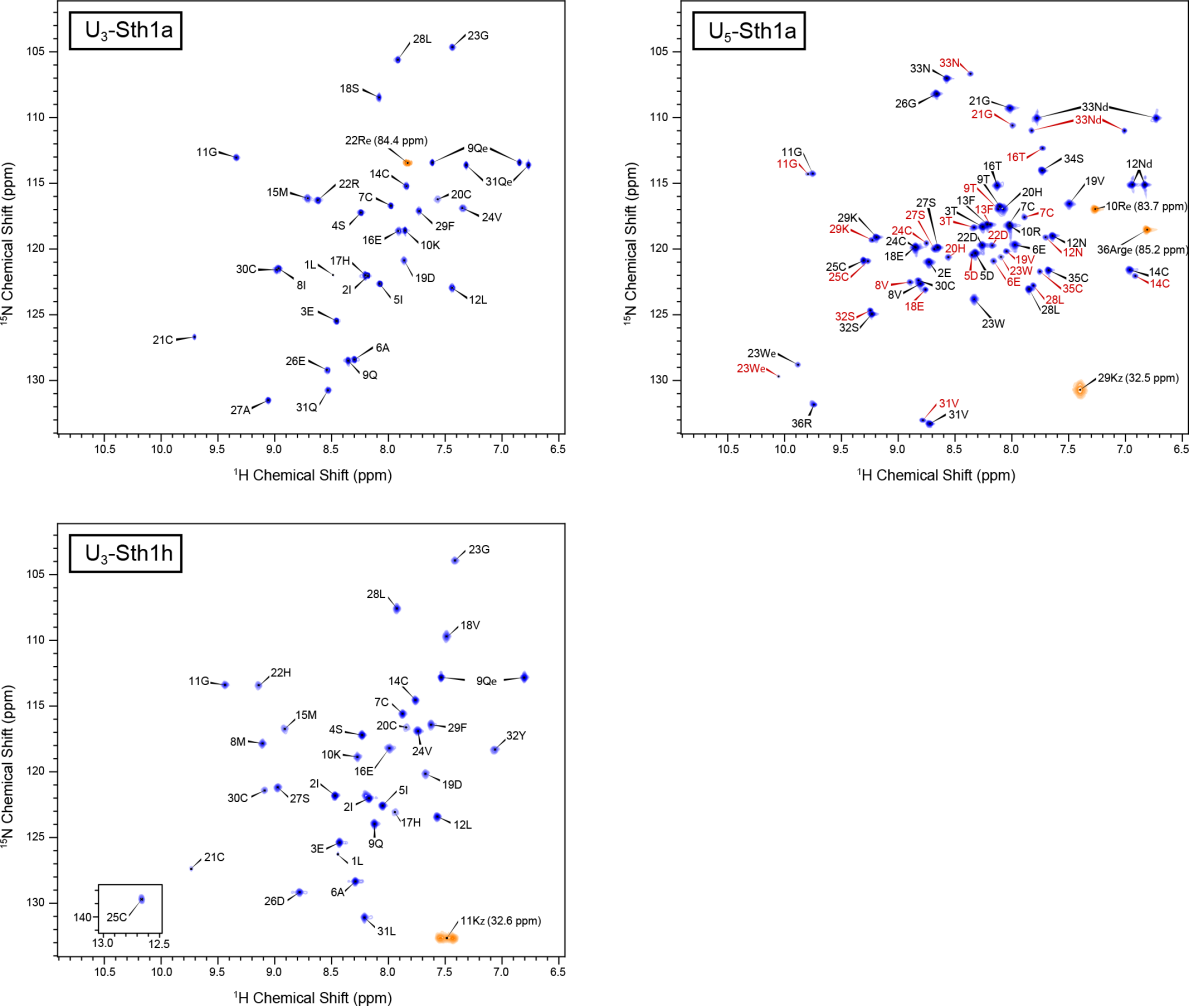
^**15**^**N-HMQC spectra of U**_**3**_**-Sth1a (top left), U**_**3**_**-Sth1h (bottom left), and U**_**5**_**-Sth1a (top right).** Sequence-specific residue assignments are indicated. Peaks from arginine and lysine side chains that were folded into the spectrum are shown in orange along with their ^15^N chemical shifts (in parentheses). The U_5_-Sth1a spectrum includes several peaks from a minor conformation; assignments for these peaks are shown with red labels.

NMR spectra were acquired at 600 MHz for ^1^H on a Bruker NMR spectrometer with a room temperature probe. The sample temperature for all NMR experiments was 298 K. Band-selective excitation short transient [27,28] (BEST) variants of the standard triple resonance sequences (HNCO, HN(CA)CO, HNCACB, HN(CO)CACB) were used for backbone assignment. ^15^N TOCSY-HSQC and HCCH-TOCSY spectra were used for side chain assignments. 2D NOESY spectra and 3D NOESY-HSQC spectra with simultaneous evolution of ^13^C and ^15^N chemical shifts were acquired for generating distance constraints. We processed the spectroscopic data using TopSpin 2.1 (Bruker Biospin) and interpreted it using Analysis 2.4.2 [29] (Collaborative Computing Project for NMR). Representative ^15^N HMQC spectra for the three peptides studied are shown in Fig 3.

### Structure Calculation

**Fig 4:**
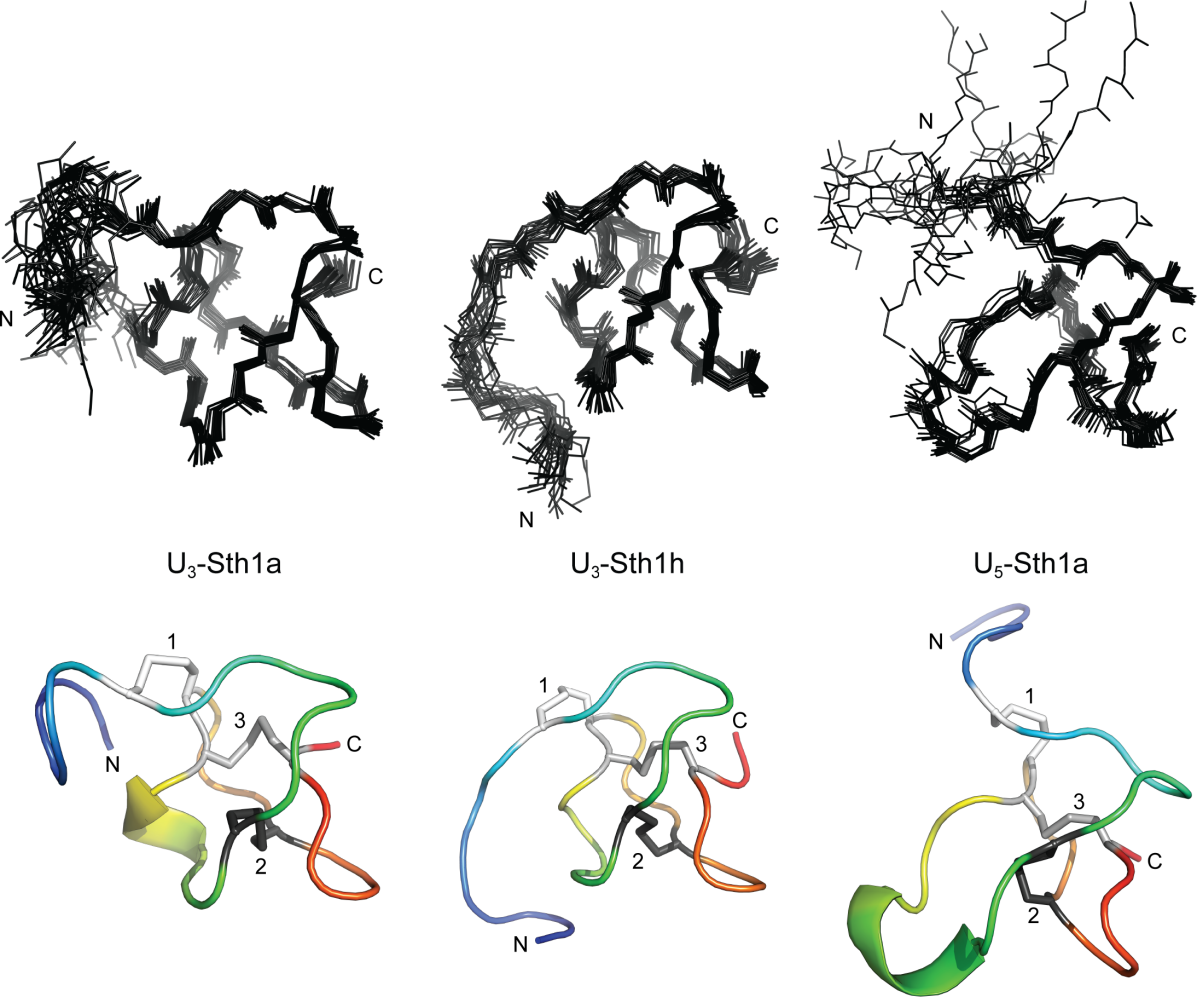
**Ensemble (top) and cartoon (bottom) representations of U**_**3**_**-Sth1a (left), U**_**3**_**-Sth1h (center), and U**_**5**_**-Sth1a (right).** The N and C termini are labeled in both the ensemble (top) and cartoon (bottom) representations of the structures of U_3_-Sth1a (left), U_3_-Sth1h (center), and U_5_-Sth1a (right). The three disulfide bonds in the cartoon representations are also labeled. The same orientation is used for the top and bottom representations. The ensembles are comprised of the 20 structures with the lowest total energy out of 100 calculated structures. The cartoon representations show the lowest energy structure from each ensemble. The N-terminal region shows some disorder for all three structures, but is especially apparent for residues 1–6 of U_5_-Sth1a.

We used chemical shift-matched peak lists from the NOESY spectra along with torsion angle restraints derived using TALOS-N [30] as input for ARIA 2.3 [31]. Disulfide-bond constraints were used in later rounds of structure calculations once the cysteine connectivities were unambiguously determined from previous iterations. We deviated from the standard ARIA protocol by using a log-harmonic potential [32], by increasing the number of cooling steps from 5000 to 15,000 for the first simulated annealing period (cool1) and from 4000 to 12,000 for the second (cool2), and by calculating 30 structures (rather than 20) for each iteration. In the final iteration, 100 structures were calculated. The 20 structures with the lowest energy were selected for water refinement, and then used to generate the structural ensembles shown in Fig 4.

The number and type of restraints used for the calculations, as well as the statistics for the structures, are provided in Table 2. As noted in Table 1, we deposited chemical shifts and restraints at the BioMagResBank (accession numbers 26002, 26003, and 26004 for U_3_-Sth1a, U_3_-Sth1h, and U_5_-Sth1a, respectively) and atomic coordinates in the Worldwide Protein Data Bank (accession codes 5FZV, 5FZW, and 5FZX for U_3_-Sth1a, U_3_-Sth1h, and U_5_-Sth1a, respectively).

**Table 2:** Structural Statistics for the U_3_-Sth1a, U_3_-Sth1h, and U_5_-Sth1a ensembles.

			U_3_-Sth1a	U_3_-Sth1h	U_5_-Sth1a
Physical Parameters (including nonnative N-terminal residues)					
	Number of residues		32	33	36
	Average molecular weight (unlabeled, kDa)		3458.1	3619.3	3920.3
	Monoisotopic molecular weight (reduced, unlabeled, Da)		3455.5	3616.5	3917.6
Structural Restraints					
	NOE-derived distance restrains (ARIA cycle 8)				
		Intraresidue (| *i*–*j* | = 0)	222	229	152
		Sequential (| *i*–*j* | = 1)	62	82	64
		Short (2 ≤ | *i*–*j* | ≤ 3)	10	18	12
		Medium (4 ≤ | *i*–*j* | ≤ 5)	8	20	11
		Long (| *i*–*j* | > 5)	43	56	32
		Ambiguous	100	104	55
		Total	445	509	326
	Dihedral constraints				
		Phi	20	25	27
		Psi	20	25	27
	Sγ-Sγ distance restraints		3	3	3
Statistics for accepted structures					
	Accepted structures		20	20	20
	Mean CNS energy terms				
		*E* total (kcal mol^–1^ ± SD)	–830 (±63)	–625 (±47)	–878 (±39)
		*E* van der Waals (kcal mol^–1^ ± SD)	–97 (±9)	–90 (±14)	–81 (±9)
		*E* distance restraints (kcal mol^–1^ ± SD)	157 (±8)	261 (±16)	163 (±9)
	Restraint violations > 0.3 Å (average # per structure)		2.9 (±1.3)	6.0 (±2.1)	4.4 (±1.4)
	RMS deviations from the ideal geometry used within CNS				
		Bond lengths (Å)	0.0034	0.0038	0.0035
		Bond angles (°)	0.54	0.71	0.54
		Improper angles (°)	1.37	3.19	1.62
Ramachandran Statistics (PROCHECK 3.5.4, [49])					
	Most favored (%)		74.8 (±3.9)	74.5 (±3.3)	77.1 (±3.8)
	Additionally allowed (%)		20.2 (±4.7)	22.7 (±4.4)	18.6 (±3.8)
	Generously allowed (%)		3.3 (±2.9)	1.4 (±2.1)	3.8 (±1.6)
	Disallowed (%)		1.7 (±1.9)	1.4 (±2.7)	0.5 (±1.7)
Average atomic RMS deviations from average structure (±SD)*					
	N, C_α_, C, and O atoms (all residues, Å)		1.40 (±0.64)	0.70 (±0.15)	2.93 (±0.76)
	All heavy atoms (all residues, Å)		1.66 (±0.59)	1.06 (±0.16)	3.36 (±0.74)
	N, C_α_, C, and O atoms (for residues with cop ≥ 0.9, Å)		0.32 (±0.09)	0.47 (±0.08)	0.48 (±0.24)
	All heavy atoms (for residues with cop ≥ 0.9, Å)		0.77 (±0.10)	0.78 (±0.10)	0.98 (±0.40)
MolProbity analyses (v3.19, [50])					
	Clashscore		12.9 (±4.1)	21.8 (±4.7)	12.1 (±4.5)
	Clashscore percentile (%)		59 (±16)	30 (±10)	63 (±19)
	Clashscore Z-score		–0.32 (±0.66)	–0.51 (±0.30)	0.40 (±0.54)

*Two sets of atomic RMS deviations are provided. The first set is for the full peptide (residues 0–31 for U_3_-Sth1a, 0–32 for U_3_-Sth1h, and 1–36 for U_5_-Sth1a) whereas the second set is calculated only including residues for which the circular order parameters (cop) for both φ and ψ are ≥ 0.9 (residues 8–30 for U_3_-Sth1a, residues 2 and 6–32 for U_3_-Sth1h, and residues 8–35 for U_5_-Sth1a).

### Functional Analysis of Recombinant Toxins

Crickets (*Acheta domestica*) were injected with up to 2 μL of recombinant peptide solution with a concentration of 1 mg/mL (U_3_-Sth1a and U_3_-Sth1h) or 2 mg/mL (U_5_-Sth1a). Crickets were then observed every 10 min for 1 h (and once again after 24 h) to determine if there was a change in how quickly the crickets were able to right themselves after being flipped over. Although a change in the righting response was noted for some crickets, the number of affected crickets was not significantly different from crickets in a control group that were injected with insect saline (data not shown).

A single high dose of each peptide dissolved in 3.4 μL of water was injected laterally into the thorax of adult blowflies (*Lucilia cuprina*; weight of 26.0–27.9 mg) as previously described [33]. The doses used for these injections were 350 nmol of U_3_-Sth1a per gram of body weight, 290 nmol/g for U_3_-Sth1h, and 310 nmol/g for U_5_-Sth1a. All flies were then kept individually in 2 mL tubes and observed at 0.5, 1 and 24 h post-treatment for signs of paralysis or lethality.

Fluorescent Ca^2+^ assays in neuroblastoma cells were performed using a fluorescent imaging plate reader (FLIPR^Tetra^, Molecular Devices, Sunnyvale, CA) as previously described [34]. SH-SY5Y cells plated on black-walled 384-well imaging plates (Corning) were loaded with Calcium 4 no-wash dye (Molecular Devices) for 30 min to assess inhibition of responses mediated by Na_v_1.7 voltage-gated sodium channels (stimulation by veratridine (4 μM) in the presence of OD1 (30 nM)), Ca_V_1.3 voltage-gated calcium channels (stimulation by KCl (90 mM)/CaCl_2_ (5 mM)), homomeric α7 (stimulation by choline (30 μM) in the presence of PNU120596 (10 μM)), and heteromeric α3-containing nicotinic acetylcholine receptors (stimulation by nicotine (30 μM)). The assays were performed in PSS (physiological salt solution, pH 7.4) containing 140 mM NaCl, 11.5 mM glucose, 5.9 mM KCl, 1.4 mM MgCl_2_, 1.2 mM NaH_2_PO_4_, 5 mM NaHCO_3_, 1.8 mM CaCl_2_, and 10 mM 4-(2-hydroxyethyl)-1-piperazineethanesulfonic acid (HEPES). The peptides (final concentration 10 μM) caused a small, but probably not functionally relevant, effect on all four channels as summarized in Table 3.

**Table 3:** Assay conditions and effect of U_3_-Sth1a and U_5_-Sth1a on Ca_V_1.3, Na_v_1.7, α7 nAChR, and α3 nAChR responses.

Target	Assay Buffer	Agonist	Control Response	U_3_-Sth1a (10 μM)	U_5_-Sth1a (10 μM)
Ca_V_1.3	PSS	KCl (90 mM) + CaCl_2_ (5 mM)	1.19	0.84	0.92
Na_v_1.7	PSS + OD1 (30 nM)	veratridine (4 μM)	0.51	0.55	0.51
α7 nAChR	PSS + PNU120596 (10 μM)	choline (30 μM)	1.00	1.07	0.84
α3 nAChR	PSS	nicotine (30 μM)	0.42	0.46	0.38

Samples of all three venom peptides were sent to the National Institute of Mental Health (NIMH) Psychoactive Drug Screening Program (PDSP) [35] for activity testing. The purpose of the PDSP is to screen potentially psychoactive compounds by testing them for activity on human and rodent central nervous system (CNS) receptors and transporters. This screen used radioligand binding assays to test for activity (i.e., inhibition or activation) against the 45 CNS targets listed in Table 4 including 11 serotonin receptors, nine adrenegic receptors, five dopamine receptors, five muscarinic acetylcholine receptors, four histamine receptors, three neurotransmitter transporters, three opioid receptors, and two sigma receptors. The PDSP tested U_3_-Sth1a, U_3_-Sth1h, and U_5_-Sth1a in quadruplicate against each of these targets, but all three failed to elicit a significant level of inhibition or activation of any of the targets.

**Table 4:** Psychoactive Drug Screening Program Targets. Names of receptors follow the International Union of Basic and Clinical Pharmacology (IUPHAR) nomenclature. Targets are cloned human receptors and transporters unless otherwise noted.

5-Hydroxytryptamine Receptors	Adrenergic Receptors	Dopamine Receptors	Muscarinic Receptors	Histamine Receptors	Neurotransmitter Transporters	Opioid Receptors	Sigma Receptors	Other Receptors
5-HT_1A_	α_1A_	D_1_	M_1_	H_1_	DAT	δ (DOR)	σ1 (guinea pig)	GABA/PBR (rat brain)
5-HT_1B_	α_1B_	D_2_	M_2_	H_2_	NET	κ (KOR)	σ2 (PC12)	GABA_A_ (rat brain)
5-HT_1D_	α_1D_	D_3_	M_3_	H_3_	SERT	μ (MOR)		GABA_A_/BZP (rat brain)
5-HT_1E_	α_2A_	D_4_	M_4_	H_4_				
5-HT_2A_	α_2B_	D_5_	M_5_					
5-HT_2B_	α_2C_							
5-HT_2C_	β_1_							
5-HT_3_	β_2_							
5-HT_5A_	β_3_							
5-HT_6_								
5-HT_7A_								

## Results and Discussion

The full prepropeptide sequences for U_3_-Sth1a, U_3_-Sth1h, and U_5_-Sth1a derived from a *Scytodes thoracica* venom-gland cDNA library are shown in Fig 1. The full sequences match the pattern expected for small peptides from spider venoms, with a hydrophobic signal sequence and a short propeptide sequence with a PQM motif preceding the mature toxin sequence. For both U_3_-Sth1a and U_3_-Sth1h, the mature toxin sequences match the consensus sequence for the ICK motif provided in the introduction. In the case of U_5_-Sth1a, the mature toxin sequence nearly fits the ICK consensus sequence albeit with one additional amino acid between the second and third cysteines. In addition, all three sequences follow the CX_3_GX_2_C motif between the first and second cysteines that is commonly found in venom peptide toxins from theraphosid and ctenid spiders. Consistent with the propeptide cleavage site we predicted based on the PQM motif, previous work [15] using mass spectrometry confirmed that U_5_-Sth1a (as well as a paralog of U_3_-Sth1a and U_3_-Sth1h) is present in crude *Scytodes thoracica* venom.

**Fig 5:**
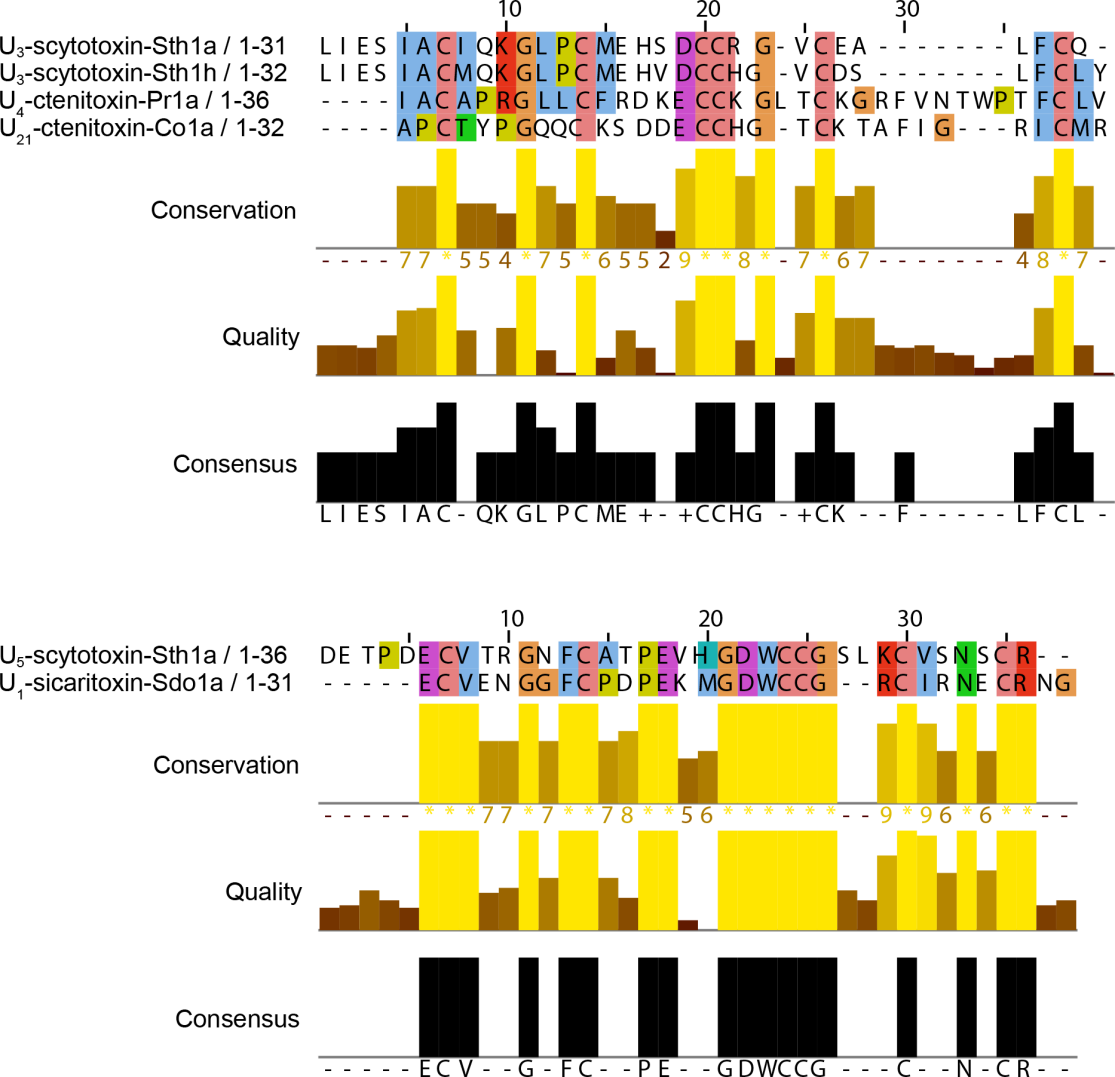
Sequence alignment for the mature toxin sequences. Alignment of the mature toxin sequences for U_3_-Sth1a and U_3_-Sth1h (top) of U_5_-Sth1a (bottom) with their closest matches to venom peptides from different species found in the ArachnoServer toxin peptide database using BLASTp. Sequences were aligned using ClustalX 2.1 and visualized using JalView 2.8.1. The coloring makes use of the default ClustalX color scheme, which is a function of sequence identity and amino acid type.

We used BLASTp [36] to search for mature toxin sequences in the ArachnoServer database [4] that align closely with the mature toxin sequences for U_3_-Sth1a, U_3_-Sth1h, and U_5_-Sth1a. Except for other sequences from *Scytodes thoracica*, no sequences matched U_3_-Sth1a or U_3_-Sth1h with expect values less than 10^−5^ (a commonly used threshold for sequence homology; [37]). For example, the closest match for U_3_-Sth1a from another species was U_4_-ctenitoxin-Pr1a, a peptide from the venom of the spider *Phoneutria reidyi* that moderately inhibits L-type voltage-gated calcium channels (Ca_V_1/CACNA1) [38] matched with an expect value of 0.009. For U_3_-Sth1h, the closest match from another species was U_21_-ctenitoxin-Co1a from the venom of the spider *Ctenus ornatus* which has an unknown molecular target [4], with an expect value of 0.016. In both cases, the main residues that align are the cysteines and a few other residues, as shown in Fig 5.

There was one significant match for U_5_-Sth1a from another species; the peptide U_1_-sicaritoxin-Sdo1a [26] from the venom of *Sicarius dolichocephalous* has an unknown molecular function and matched with an expect value of 2×10^−8^. Potentially, this match may stem from the evolutionary relationship between *Sicarius dolichocephalous* and *Scytodes thoracica* as it has been proposed that their families (Sicariidae and Scytodidae, respectively) are sister taxa [39] that diverged at least 100 million years ago [40].

The structures for U_3_-Sth1a, U_3_-Sth1h, and U_5_-Sth1a determined using NMR spectroscopy are shown in Fig 4 as an ensemble of 20 structures (top) and a cartoon representation of the lowest energy structure (bottom). As predicated based on their sequences and origin, all three structures were found to contain an ICK motif, with disulfide bonds 1, 2, and 3 formed by cysteines I and IV, II and V, and III and VI, respectively. For all three peptides, the β-hairpin between cysteines V and VI is unusually truncated and, in the case of U_5_-Sth1a, the region between cysteines II and III is longer than that found in most other venom toxins.

When expressing proteins and peptides with multiple disulfide bonds there is the potential for these bonds to not form or to form with the incorrect topology. This is particularly important in cases, such as this one, where the peptides have not been functionally characterized as we do not have assays to determine whether the structures that we observed are functionally relevant. Nevertheless, there are several reasons why we think our structures represent the native folds. First, it has been shown repeatedly that spider venom toxins are able to fold correctly *in vitro* [41–43]. Second, the connectivity of the cysteines in our structures follow the pattern that we expected based on comparison with known spider venom toxins. Third, the chemical shifts for the cysteine α and β carbons [44] in our NMR spectra are consistent with cysteines in disulfide bonds (i.e., completely oxidized) and inconsistent with reduced cysteines. Finally, testing of our peptide samples with Ellman's Reagent (5,5’-dithiobis(2-nitrobenzoic acid), DTNB) [45] indicated that there are no reduced cysteines present. Taken together, these data indicate that the cysteines in U_3_-Sth1a, U_3_-Sth1h, and U_5_-Sth1a are fully oxidized and connected with the expected topology, leading us to believe that these peptides are correctly folded.

The ^15^N HMQC spectrum for U_5_-Sth1a (Fig 3, lower spectrum) reveals the presence of a minor conformation of this peptide. The minor conformation must have very similar surface properties to the major conformation as we could not separate the two conformations using liquid chromatography. In addition, the presence of a minor component is probably not due to proteolysis as we did not observe a secondary peak in electrospray ionization mass spectra (Fig 2). As the chemical shifts of the minor conformation in the ^15^N HMQC spectrum differ only slightly from those of the major conformation U_5_-Sth1a, it must adopt a very similar fold. One possibility is that the minor conformation corresponds to U_5_-Sth1a with incomplete disulfide bond formation. However, the chemical shifts for the cysteine α and β carbons of the minor conformation are consistent with all of the cysteines being oxidized (and therefore in disulfide bonds). Another possibility is that the minor conformation contains disulfide bonds in a different topology (i.e., between cysteines II and VI, and III and V). This could happen with very little distortion of the overall structure as these cysteines are close to one another. To test this hypothesis, we mapped the amide chemical shift differences between the major and minor conformations onto the structure. We found that the largest changes are in the part of the structure with the short α-helical region (residues 16–23), whereas the amide chemical shifts for the cysteines that would have non-native connections (14C, 24C, 30C 35C) change only modestly. This leads us to believe that both conformations have the expected disulfide bonding pattern, and that the difference is due to conformational exchange in the region of residues 16–23 that is slow on the NMR timescale.

The conserved pattern of cysteines, the presence of the ICK structural motif, and the venom gland source of U_3_-Sth1a, U_3_-Sth1h, and U_5_-Sth1a leads us to believe that these peptides are likely to be neurotoxins. Evolution has fine-tuned a majority of ICK peptides from spider venoms to target insect ion channels. While some ICK peptides evolved to target mammalian channels [46], a large majority have not, so in many cases any interaction in mammals is due to structural homology of the ion channel. Unfortunately, our attempts so far at functional characterization have not allowed us to determine the targets of the three *Scytodes* peptides. That the peptides failed to elicit a significant level of activation or inhibition of 45 different human and rodent CNS targets, as tested by the PDSP, is not surprising, as this screen did not involve targets from insects and the targets were mainly receptors rather than ion channels.

Injection of high doses of U_3_-Sth1a (350 nmol/g) and U_5_-Sth1a (310 nmol/g) into sheep blowflies did not cause any paralytic or lethal activity. Likewise, injection of doses of up to 6 nmol/g (U_3_-Sth1a and U_3_-Sth1h) and 12 nmol/g (U_5_-Sth1a) into crickets did not cause any paralytic or lethal activity. The lack of response when injected into crickets and blowflies is significant, but a likely explanation is that the particular peptides in this study have a high specificity for targets in prey species that are evolutionarily distant from the ones that we have studied so far.

A more remote possibility is that the peptides that we have studied are no longer functionally relevant. Spider venoms are cocktails that contain hundreds of components, the majority of which are ICK peptides. The large number of ICK peptides in the venom from individual species has evolved due to a combination of gene duplication events and focal hypermutation of the intercysteine regions [12,47, 48]. The peptides from the venom of a single species of spider vary considerably in target and species-specificity. The complexity of the venom cocktail allows spiders to paralyze a wide variety of prey species, but it also means that loss-of-function mutations of individual peptides might be tolerated. However, the genes for non-functional peptides would no longer be under selective pressure and would eventually mutate to sequences that would not be expressed or that would not be recognizable as spider venom peptides. As the sequences and structures for U_3_-Sth1a, U_3_-Sth1h, and U_5_-Sth1a follow the patterns that we expect for ICK venom peptides from spiders, and evidence exists that U_5_-Sth1a and at least one paralog of U_3_-Sth1a/U_3_-Sth1h are present in crude venom [15], we feel that the most parsimonious explanation for our inability to observe any biological of activity for these peptides is that they are functional but their molecular targets remain elusive.

## Conclusion

We have presented the first three-dimensional structures for spider-venom peptides from the Scytodidae family. These peptides are structurally similar to other venom peptides in that they contain an ICK structural motif, but show little sequence identity to peptides from other species of spiders. Although the targets of these peptides remain elusive, we hope that further study will help determine their functions.
